# Effects of positive and negative childhood experiences on adult family health

**DOI:** 10.1186/s12889-021-10732-w

**Published:** 2021-04-05

**Authors:** Chantel L. Daines, Dustin Hansen, M. Lelinneth B. Novilla, AliceAnn Crandall

**Affiliations:** Department of Public Health, Brigham Young University, 4103 LSB, Provo, UT 84602 USA

**Keywords:** Adverse childhood experiences, Positive childhood experiences, Family health, Resilience, Life-course theory

## Abstract

**Background:**

The objective of the study was to determine the association between adverse childhood experiences (ACEs) and positive childhood experiences (PCEs) with family health in adulthood. Prior research indicates that ACEs and PCEs affect individual physical and mental health in adulthood. However, little is known about how ACEs and PCEs affect family health. Families develop and function through patterns and routines which are often intergenerational. Therefore, a person’s early experiences may influence their family’s health in adulthood.

**Method:**

A survey was administered to 1030 adults through Qualtrics, with participants recruited using quota-sampling to reflect the demographic characteristics of U.S. adults. Participants completed a survey about their childhood experiences, four domains of family health (family social and emotional health processes, family healthy lifestyle, family health resources, and family external social supports), and demographic characteristics. Data were analyzed using structural equation modeling.

**Results:**

After controlling for marriage, education, gender, race and age, ACEs were negatively associated with family social and emotional health processes and family health resources when accounting for PCEs; PCEs were positively associated with all four family health domains irrespective of ACEs.

**Conclusion:**

Childhood experiences affect family health in adulthood in the expected direction. Even in the presence of early adversity, positive experiences in childhood can provide a foundation for creating better family health in adulthood.

## Background

Family health is “a resource at the level of the family unit that develops from the intersection of the health of each family member, their interactions and capacities, as well as the family’s physical, social, emotional, economic, and medical resources” [1]. Family health is shaped not only by genetics but also by family functioning and family experiences, including the ability of the family to adapt to internal and external challenges and experiences. Positive family health promotes belonging, caring, and the capacity to perform family responsibilities, which in turn, promotes the health of individual members [2].

Family health includes a variety of factors that transcend disciplinary boundaries, including family communication and problem-solving, family functioning and routines, emotional support, healthy behaviors, internal coping skills of the family, and access to external resources [1, 3]. Key domains of family health include: (1) family social and emotional health processes; (2) family healthy lifestyle; (3) family health resources; and (4) family external social support [3]. Family social and emotional health processes include family relationships, communication, support, and feelings of emotional safety and belonging that promote cohesion within the family. This aspect of family health aligns most closely with traditional measures of family functioning [3]. Family healthy lifestyle comprises a family’s collective healthy choices through regular physical activity, eating fruits and vegetables, following doctor’s recommendations, and seeking health care services as needed. Family health resources refer to financial and non-material assets that allow the family to carry out their functions and their normal daily activities. These include internal and external resources such as help-seeking efficacy, the ability to effectively cope with family challenges, financial resources and other basic needs, and access to health care. Finally, family external social supports refers to the presence of a social network or social capital outside of the family that the family can count on for advice, care, or assistance, whether financial or otherwise [3]. These domains offer protection against many physical and mental issues within families by creating stronger relationships and family functioning [4–8].

The measurement of family health is relatively new [1–3], and understanding how one’s family experiences in childhood affects family health in adulthood is not currently well understood. Therefore, in an effort to better understand the intergenerational transmissibility of family health, in this study we examine the effects of retrospectively reported adverse childhood experiences (ACEs) and positive childhood experiences (PCEs) that occurred before age 18 years on family health in adulthood. ACEs are potentially traumatic life events that occur before the age of 18, including child abuse (physical, emotional or sexual) and neglect, mental illness of family members, parental divorce or separation, and family member substance use [9]. PCEs (also referred to as benevolent childhood experiences (BCEs) or advantageous childhood experience (counter-ACEs) in other studies [10, 11]), are experiences before age 18 that are thought to be beneficial, such as positive relationships with parents and other adults, household routines, beliefs that provide comfort, and having good neighbors.

### Childhood experiences and later health: resiliency and life course theories

Resiliency Theory and the Life Course Theory provide the theoretical framework for this study. Resiliency Theory focuses on promotive factors that independently lead to better health and may attenuate the effects of risk factors on developmental trajectories and outcomes [12]. Promotive factors allow for post-traumatic cognitive and social growth [13], which can equip individuals with coping skills, self-worth, self-efficacy, and optimism [14]. By applying Resiliency Theory to the current study, PCEs can be viewed as promotive factors that include healthy interactions with mentors, role models, and positive influences. In addition to internal skills, PCEs enable the individual to develop resilient functioning and continued growth even when traumatic events occur [13–16]. This resiliency and growth my help to promote better family health in adulthood.

Life Course Theory [17] offers a comprehensive intergenerational approach for understanding how the timing of life events, especially during sensitive developmental periods, have biopsychosocial consequences that could alter health trajectories [17–19], including family health. Human development, as viewed through the Life Course Theory, is comprised of interconnected biological changes at various life stages that interact with psychosocial factors over time. Thus, early experiences such as separation, family dysfunction, neglect, abuse, violence, and resource restrictions generate delayed pathology that influence subsequent health, including access to care and parental resilience [9, 18]. Where early adversity produces “distinct patterns of disadvantage or privilege” over time [18], having more positive childhood experiences allows for improved wellbeing in adulthood [14]. Forrest & Riley [20] attribute such effects on biopsychosocial processes that permit successful adaptation despite negative stressors.

Three key principles of the Life Course Theory provide a framework for understanding how childhood experiences can affect family health in adulthood: linked lives, human agency, and lifelong development and aging [17]. “Linked lives” is the interdependence of shared relationships and experiences that are most apparent in the family setting. For instance, ACEs and PCEs are strongly influenced by the circumstances in the family. Families that communicate well and do things together often facilitate healthy relationships for children, leading to more PCEs. On the other hand, families with fewer resources may have more difficulty in building healthy relationships and may have fewer coping resources. Thus, when stressors arise, family abuse and dysfunctionality may arise leading to more ACEs. ACEs and PCEs trigger a series of biopsychosocial mechanisms. ACEs can result in HPA axis dysfunction and chronic inflammation [21], with the effects of the initial trauma manifesting in adulthood in a variety of disorders and poor health outcomes, whereas PCEs can lead to adaptation and resilience [16, 22]. These early life events may work independently or together in affecting the quality of family health an individual is able to create in adulthood. On the other hand, “human agency” emphasizes how individuals and families can make choices in the presence of opportunities, constraints, and/or adversity. For example, even when families experience stress and trauma, they can still create safe, trusting relationships and stable routines for their children. Finally, “lifelong development and aging” is examining individual human development in terms of interconnected stages of physical, mental, social, and spiritual changes throughout the life span. For instance, the consequences of ACES that are transmitted across generations may be tempered by various factors over time such as the presence of PCEs.

### Childhood experiences and family health in adulthood

Prior research has demonstrated that childhood experiences affect individual health in adulthood. For example, individuals who experience numerous ACEs early in their childhood are at risk for developing depression, anxiety, substance abuse habits, and detrimental health behaviors as they mature into adulthood [23]. Conversely, PCEs independently lead to better health and may offset the effects of ACEs on adult health [10, 11, 24, 25].

Although research on childhood experiences indicates their influence on adult health, little is known about how ACEs and PCEs affect family life. Research has demonstrated that parenting styles are often passed down to children who then parent their children using similar methods and traditions [26, 27]. Families develop through family patterns and life cycle events and processes [28]. For example, research indicates that individuals who were abused as children are more likely to abuse their own children [27], and parents who have unresolved emotional issues from childhood are more disorganized in their parent child attachments and exhibit more frightening parenting behaviors [8, 29]. On the other hand, parents that were raised in a home that had more positive coping strategies and parenting efficacy were more likely to perpetuate these positive coping strategies in their own families [26]. Other studies have shown that parents who have experienced more PCEs in their childhood are better able to provide a positive home life for their children [26] and have improved family function, family cohesion and overall health [5, 30, 31].

### Aims and hypotheses

In this study, we examined the effects of ACEs and PCEs on a comprehensive measure of adult-reported family health. The aim of the study was to determine the association between childhood experiences, both adverse and advantageous, and family health in adulthood. We hypothesized that (1) ACEs would lead to worse family health outcomes across all four domains of family health; and (2) PCEs would improve family health across all domains. (3) Given that prior research has shown that PCEs have a direct positive effect on individual health irrespective of ACE score, and that the effects of ACEs on health is attenuated when PCEs are included in models [10], we also hypothesized that PCEs would have a stronger association with family health than ACEs.

## Methods

### Participants and procedures

A cross-sectional sample of 1030 individuals, ages 18 years or older, was recruited from a Qualtrics panel. Qualtrics manages a proprietary panel of members who are available for research. In addition to age, participants were recruited using demographic quotas for low education, minority race, being born outside of the U.S., and family structure (e.g. married, single, households with children, empty nesters, etc.). The quotas were based on estimates from the U.S. Census to ensure a sample that was varied based on family structure and demographically representative of the U.S. population. Each participant was asked to answer a 115-item survey about ACEs, PCEs, and family health. The survey took approximately 10–15 min to complete. The study was approved by the university’s Institutional Review Board (IRB).

### Measures

#### Family health

Family health was measured using the 32-item Family Health Scale (FHS) [3]. The FHS is a holistic measure of family health that was developed based on two theoretical frameworks: the Denham Family Health Framework [4] and the Family Health Measurement Resource Network Model [1]. Based on these frameworks, the FHS includes the following four subscales: family emotional and social health processes (α = .92), family healthy lifestyle (α = .87), family health resources (α = .82), and family external social supports (α = .85). Response options were on a 5-point Likert scale ranging from 1 (strongly disagree) to 5 (strongly agree). Higher scores indicated better family health. Participants were asked to answer each question based on who they considered to be their family. Sample items from each of the four family health domains included: “In my family, I feel safe in my family relationships” (family social and emotional health processes), “In my family, we help each other avoid unhealthy habits” (family healthy lifestyle), “In my family, we have people outside of our family we can turn to when we have problems at school or work” (family external social supports), and “In my family, a lack of health insurance would prevent us from asking for medical help” (family health resources).

#### Childhood experiences

Adverse childhood experiences were measured using the 11-item ACE module from the Centers for Disease Control and Prevention’s Behavioral Risk Factor Surveillance System Survey [32]. Responses were dichotomized (0 = ACE did not occur; 1 = ACE was experienced). Three ACEs were related to sexual abuse and those items were combined into one ACE item, with a yes on any of the three items leading to a confirmative ACEs. The ACE items were summed for a final ACEs score ranging from 0 to 9. Each question included the following stem “When you were growing up, during your first 18 years of life …” Sample items included: “Did a parent or other adult in the household often swear at you, insult you, put you down, or humiliate you?” “Did you live with anyone who was a problematic drinker or alcoholic or who used street drugs?” and “Were your parents ever separated or divorced?”

Positive childhood experiences were measured using the 10-item Benevolent Childhood Experiences scale [11]. For each question, respondents reported whether the event occurred before age 18 years. The total number of “yes” responses were summed, for a final PCE score ranging from 0 to 10. Higher scores indicated more PCEs. Sample items included: “Did you have a predictable home routine, like regular meals and a regular bedtime?” “Did you have beliefs that gave you comfort?” and “Was there an adult who could provide you with support or advice?”

#### Controls

Given that one’s experience of family life may vary based on their marital status (married = 1; non-married = 0), age (in years), and gender (female = 1; male = 0), we included those variables as covariates in the model. For example, participants who were married may have viewed their family as their spouse and children while an unmarried participant may have been more likely to consider family members outside of their nuclear family. Likewise, expectations about family roles and life may vary based on participant generational effects (e.g., boomers vs millennials) or gender [33]. Prior research indicates that minority races and those with lower SES have higher risk for ACEs [34, 35]. To account for this in our models, we also controlled for participant race (white = 1; non-white = 0) and education (bachelor’s degree = 1; less than bachelor’s degree = 0).

#### Analytic methods

Data were cleaned in Stata 16. Structural equation modeling (SEM) was used to estimate model paths using Mplus version 7. SEM is a valuable analytic tool because it allows one to control for measurement error and to examine multiple pathways simultaneously. Given that prior research has shown that the four family health outcomes are correlated [3], examining multiple pathways simultaneously was important for this analysis so that we could assess all of family health together rather than separately. Confirmatory factor analysis (CFA) was conducted to establish the measurement model. The four family health subscales were included as latent variables. Next, a structural model was fit by regressing the four family health latent variables on PCEs and ACEs. Controls were included in the model by regressing the independent and dependent variables on gender, age, education, marital status, and race. Model fit for the measurement and structural models was estimated using the following model fit indices and cutoffs: Root Mean Square of Approximation (RMSEA) < .06 and Comparative Fit Index (CFI) > .90 (30). Robust weighted least squares maximum likelihood, appropriate for categorical data, was used to estimate the final model. Missing data were minimal (no more than 0.49% on any variable) and handled using full information maximum likelihood.

## Results

Participants had 2.6 ACEs on average; the average PCEs score was 8.2. Most of the sample, 60.8%, were non-Hispanic white, and the average age was 40.4 years; 53.4% percent of participants were female and 46.2% were married; 35.3% had a bachelor’s degree and 11.9% had less than a high school diploma. Table 1 includes the descriptive statistics for the sample.

**Table 1:** Descriptive statistics of the sample, *N =* 1030

	%
Age, *M* (SD)	40.4 (17.3)
Married	46.2
People per household, *M* (SD)	3.2 (2.1)
Education	
Less than high school	11.9
Bachelor’s degree	35.3
Female	53.5
Non-Hispanic white	60.8

Confirmatory factor analysis using the data from this sample was conducted in a prior study [3] and demonstrated adequate model fit of the measurement model (RMSEA = .059; CFI = .958). Factor loadings ranged from .52 to .90 across the four FHS subscales.

Table 2 contains the results of the structural equation models examining ACEs and covariates only, PCEs and covariates only, and fully adjusted models with ACEs and PCEs included. In models without PCEs, ACEs were negatively associated with all four family health domains (family social and emotional health processes: −.17, *p* < .001; family healthy lifestyle: −.12, *p* < .001; family health resources: −.28, *p* < .001; family external social supports: −.07, *p* < .05). After accounting for PCEs, ACEs were negatively associated with family social and emotional health processes (−.09, *p* < .01) and family health resources (−.20, *p* < .001), but were not associated with family healthy lifestyle nor family external social supports. PCEs were positively associated with each family health domain, irrespective of ACEs score (family social and emotional health processes: .24, *p* < .001; family healthy lifestyle: .26, *p* < .001; family health resources: .25, *p* < .001; family external social supports: .31, *p* < .001). The final structural model, with four latent variables, demonstrated adequate fit (RMSEA = .05; CFI = .95; see Table 2). ACEs and PCEs were negative correlated (−.30, *p* < .001).

**Table 2:** Structural equation model of the effects of childhood experiences on family health in adulthood, *N* = 1030

	Family Social and Emotional Health Processes			Family Healthy Lifestyle			Family Health Resources			Family External Social Supports		
	ACEs Model	PCEs Model	Adjusted Model	ACEs Model	PCEs Model	Adjusted Model	ACEs Model	PCEs Model	Adjusted Model	ACEs Model	PCEs Model	Adjusted Model
ACEs	−.17***	–	−.09**	−.12***	–	−.04	−.28***	–	−.20***	−.07*	–	−.03
PCEs	–	.27***	.24***	–	.27***	.26***	–	.31***	.25***	–	.30***	.31***
**Controls**												
Female	.03	.02	.02	−.03	−.04	−.04	.03	.01	.02	−.02	−.03	−.03
Age	.00	.00	−.01	−.04	−.05	−.05	.15***	.16***	.14***	−.23***	−.24***	−.24***
Married	.20***	.19***	.18***	.18***	.16***	.16***	.07*	.06	.05	.10**	.07*	.08*
Non-Hispanic White	−.01	.00	.00	−.01	.01	.01	.09**	.10**	.10**	.02	.04	. 04
Bachelor’s degree	.10**	.09**	.08	.17***	.15***	.15***	−11**	.11**	.09**	.14***	.11**	.11**

Note: Coefficients are standardized. Adjusted model included both ACEs and PCEs along with controls

Model fit for fully adjusted model: RMSEA = .05; CFI = .95

*p < .05. **p < .01. ***p < .001

### Sensitivity analyses

Participants answered family health questions based on who they considered as their family. Participants were asked to define whether they answered questions about family health based on only people who lived in their household, only people who lived outside of their household, or a combination of both. We conducted a sensitivity analysis controlling for who participants considered to be their family when answering the FHS questions (1 = participant considered only family members who lived in their household; 0 = participant considered family members who lived outside of household). Additionally, given the salience of income to childhood experiences [34, 35], we added income as a control variable. Results did not vary substantively when adding these controls. Next, given that research shows that having four or more ACEs is particularly harmful to adult health [9], we used a dichotomous version of the ACEs score (1 = four or more ACEs; 0 = fewer than four ACEs). Results did not vary substantively from the results of the main model. Finally, given that some research has demonstrated meaningful results when examining family dysfunction ACEs separately from abuse and maltreatment ACEs [36], we created two separate ACE variables. In models including PCEs, family dysfunction ACEs, and child abuse/maltreatment ACEs, PCEs were similarly associated with all four family health domains as with the main model shown in Table 2. Family dysfunction and abuse/maltreatment ACEs were not associated with any domains of family health except for family health resources (family dysfunction ACEs: −.10, *p* < .01; abuse/maltreatment ACEs: −.13, *p* < .001). PCEs were more strongly correlated with abuse/maltreatment ACEs (−.31, *p* < .001) than with family dysfunction ACEs (−.16, *p* < .001).

## Discussion

The purpose of this study was to measure the association between childhood experiences with adult family health. Results indicated that childhood experiences, particularly PCEs, were predictive of family health in adulthood. Specifically, consistent with Hypothesis 1, ACEs were negatively associated with family social and emotional health processes and family health resources, irrespective of PCE score. However, when accounting for PCEs, ACEs were not associated with family healthy lifestyle nor with family external social supports. Conversely, regardless of ACE score, PCEs were positively associated with all four family health domains (Hypothesis 2). The absolute value of the standardized betas portraying the relationship between childhood experiences and family health was larger for PCEs as compared to ACEs across all four domains of family health (Hypotheses 3).

Given the known long-term effects of ACEs on individual health [9], the negative relationship between ACEs and family health is not surprising. Individuals with high ACE scores often struggle with the mental health issues of anxiety and depression [23, 37], which influence how an individual is able to process emotions, support others, respond to stressors, communicate, and make emotional connections [6]. These emotional and social skills affect the family, including social and emotional health processes that are so important to relationship development and accrual of resources. The inverse relationship between ACEs and family social and emotional health process may be explained by existing research showing that negative experiences in childhood adversely affect interpersonal relationships and may cause adult relationship difficulties including to existing family relationships dynamics and that of new family relationships (e.g., marriage and parenting) [8]. The current study showed that ACEs had the strongest negative association with the family health resources domain. This relationship may be explained by the fact that childhood trauma can affect an individual’s ability to maintain stable employment [38]. Unstable employment often leads to chronic financial hardships, which can translate into inadequate food, poor housing conditions, lack of transportation and no medical insurance for the family. Other possible explanations for the relationship of ACEs with worse family health in adulthood may include the endurance of unhealthy family relationships between childhood and adulthood, modeling of parenting and marriage relationships from the participant’s own childhood, and other factors associated with the intergenerational transmission of family health. Further research, using longitudinal data with diverse samples, is needed to better determine and understand the relationships between childhood experiences and later family health.

Irrespective of ACEs score, PCEs were associated with better family health. The strength of association was consistent across all of the family health domains. These findings indicate that PCEs set a positive trajectory for lifelong family health. PCEs include positive role models, emotional support, and family stability in childhood. Children that experience more emotional support from family or social networks may have better long-term mental health outcomes and less chronic health issues as they age, which are important family health resources [39]. Furthermore, individuals with high PCEs tend to have more self-confidence which provides a foundation for healthy relationships in adulthood [8, 40] and serves to buffer against mental health issues [19, 20, 25, 41]. All of these effects are resources that adults can draw upon to foster healthy routines, affection, respect, communication, trust, and support in their families and also engender healthy social support networks that the family can draw on when additional help is needed. Furthermore, the same healthy family that helped to foster PCEs during childhood likely continues to be a supportive family system in adulthood, even as family membership and structure may change through births, marriage, death, and variation in relationships through life stages.

Family health is comprised of many different aspects of health processes at the family level. This study demonstrates that family health can be influenced by an adult’s childhood experiences. As an adult, they now have responsibilities to provide, guide, and nurture the next generation while also continuing to support their family of origin. Childhood abuse and trauma may lead to weaker family social and emotional health processes and fewer family health resources in adulthood, either due to ongoing dysfunctional family relationships that have continued since childhood or due to their inability to foster new healthy family relationships and resources in adulthood as a result of earlier adversity. However, whether or not an adult experienced childhood trauma, if they had a high number of PCEs they are more likely to have healthier and more stable relationships in adulthood, greater self-confidence, and better stress management skills that help to foster healthy families [42].

The findings of the current study highlight the importance for public health professionals to consider the health of current and future families through the prevention of ACEs and promotion of PCEs. Several upstream and midstream efforts may serve as effective intervention strategies [43]. Healthcare professionals may consider implementing evidence-based home visiting (e.g., Nurse Family Partnership and SafeCare) and parenting (e.g., Triple P) programs and other family-based frameworks such as Family-Checkup and Health Outcomes from Positive Experiences (HOPE) [44, 45] that have been successful across a variety of contexts. Additionally, ACE and PCE questionnaires may be important standard screening in healthcare and social service settings. Such screening will allow healthcare professionals and service providers to actively work to connect children and families with services and resources that will help curtail family dysfunction or abuse. We caution that when using PCE and ACE measures as screening tools, that ACEs and PCEs measures do not examine the severity or frequency of each experience [46]. Rather, each experience is weighted equally in both PCE and ACE instruments. The value of using PCE and ACE questionnaires as screeners comes at being able to assess whether a child and their family may be experiencing a variety of positive or adverse events rather than pinpointing key individual events that may be hindering or promoting child wellbeing. Another potential screener of childhood family health may be using the FHS short-form, which includes a 10-item screener of overall family health [3]. However, despite preliminary encouraging results, the FHS requires further testing and validation of the screener in a variety of populations to more fully determine its efficacy as a screening tool.

This study includes limitations that are important to note. Participants reported on their childhood experiences at the same time that they reported on their adult family health, and they may not have accurately recalled childhood experiences. Additionally, this simultaneous reporting may have led to bias on how participants reported their childhood experiences based on their satisfaction with their current family life. Longitudinal research would be invaluable to more fully understand intergenerational trends in family health. However, it is important to note that despite concerns in recall bias, average ACE and PCE scores in the current study were similar to prior studies [10]. Another limitation is that only one family member reported on their family’s health. Thus, the family health measure was based on one individual experience with family health rather than the perspective of the whole family. Given that the responses were based on self-report, there may have been a degree of social desirability bias. Future research should include multiple respondents from the same family to gauge family health from the perspective of different family members. Although we used quota sampling techniques to represent a variety of family types and socioeconomic statuses, the data was not from a nationally representative sample and Qualtrics panels are convenience samples. Past research has shown that Qualtrics online panels provide reliable samples and do a good job of reaching participants in hard-to-reach areas [47, 48]. We included attention filters in the survey to help ensure response validity. Future research should examine the study relationships in nationally representative samples.

## Conclusion

This study provides preliminary data that increases understanding about how childhood experiences affect their later family health. Integrating resilience theory with the life course theory, this study indicates that both ACEs and PCEs affect family health in adulthood, though PCEs appear to be particularly salient to future family health. A breadth of positive childhood experiences in the home, community, and school may have a significant impact on future family life, even in the presence of adversity. Further research is needed to better understand the intergenerational transmission of family health using longitudinal and diverse samples. Public health professionals can apply upstream and mid-stream intervention efforts to promote PCEs and prevent ACEs in an effort to promote family health across generations.

## Data Availability

The datasets generated and/or analyzed during the current study are not publicly available due to IRB requirements but are available from the corresponding author on reasonable request.

## Abbreviations

ACE: Adverse childhood experiences
CFA: Confirmatory Factor Analysis
PCE: Positive childhood experiences
SEM: Structural Equation Modeling

## References

[1] Weiss-Laxer NS, Crandall A, Okano L, Riley AW (2020). Building a Foundation for Family Health Measurement in National Surveys: a modified Delphi expert process. Matern Child Health J.

[2] Schumann DA, Mosley WH (1994). The household production of health: introduction. Soc Sci Med.

[3] Crandall A, Weiss-Laxer NS, Broadbent E, Holmes EK, Magnusson BM, Okano L, et al. The family health scale: reliability and validity of a short- and long-form. Front Public Health. 2020;8:734. 10.3389/fpubh.2020.587125 PMC7717993 33330329

[4] Denham SA (2003). Family health: a framework for nursing.

[5] Klever P (2015). Multigenerational relationships and nuclear family functioning. Am J Fam Ther.

[6] Lovejoy MC, Graczyk PA, O'Hare E, Neuman G (2000). Maternal depression and parenting behavior: a meta-analytic review. Clin Psychol Rev.

[7] Musick K, Meier A (2012). Assessing causality and persistence in associations between family dinners and adolescent well-being. J Marriage Fam.

[8] Szepsenwol O, Simpson JA, Griskevicius V, Raby KL (2015). The effect of unpredictable early childhood environments on parenting in adulthood. J Pers Soc Psychol.

[9] Felitti VJ, Anda RF, Nordenberg D, Williamson DF, Spitz AM, Edwards V (1998). Relationship of childhood abuse and household dysfunction to many of the leading causes of death in adults: the adverse childhood experiences (ACE) study. Am J Prev Med.

[10] Crandall A, Miller JR, Cheung A, Novilla LK, Glade R, Novilla MLB, et al. ACEs and counter-ACEs: how positive and negative childhood experiences influence adult health. Child Abuse Negl. 2019;96:104089. 10.1016/j.chiabu.2019.104089. 10.1016/j.chiabu.2019.104089 31362100

[11] Narayan AJ, Rivera LM, Bernstein RE, Harris WW, Lieberman AF (2018). Positive childhood experiences predict less psychopathology and stress in pregnant women with childhood adversity: a pilot study of the benevolent childhood experiences (BCEs) scale. Child Abuse Negl.

[12] Zimmerman MA (2013). Resiliency theory: a strengths-based approach to research and practice for adolescent health.

[13] Cicchetti D, Rogosch FA (2009). Adaptive coping under conditions of extreme stress: multilevel influences on the determinants of resilience in maltreated children. New Dir Child Adolesc Dev.

[14] Skodol AE, Bender DS, Pagano ME, Shea MT, Yen S, Sanislow CA, et al. Positive childhood experiences: resilience and recovery from personality disorder in early adulthood. J Clin Psychiatry. 2007;68(7):1102–8. 10.4088/JCP.v68n0719. 10.4088/jcp.v68n0719 PMC2705622 17685749

[15] Bronfenbrenner U (1994). Ecological models of human development. Read Dev Child.

[16] Masten AS (2015). Pathways to integrated resilience science. Psychol Inq.

[17] Elder GH (1998). The life course as developmental theory. Child Dev.

[18] Burton-Jeangros C, Cullati S, Sacker A, Blane D (2015). A life course perspective on health trajectories and transitions: springer nature.

[19] Cheng TL, Johnson SB, Goodman E. Breaking the intergenerational cycle of disadvantage: the three generation approach. Pediatrics. 2016;137(6):e20152467. 10.1542/peds.2015-2467 PMC4894258 27244844

[20] Forrest CB, Riley AW (2004). Childhood origins of adult health: a basis for life-course health policy. Health Aff.

[21] McEwen BS (2013). The brain on stress: toward an integrative approach to brain, body, and behavior. Perspect Psychol Sci.

[22] Masten AS, Barnes AJ (2018). Resilience in children: developmental perspectives. Children..

[23] Chartier MJ, Walker JR, Naimark B (2010). Separate and cumulative effects of adverse childhood experiences in predicting adult health and health care utilization. Child Abuse Negl.

[24] Bethell C, Jones J, Gombojav N, Linkenbach J, Sege R (2019). Positive childhood experiences and adult mental and relational health in a statewide sample: Associations across adverse childhood experiences levels. JAMA Pediatrics.

[25] Gunay-Oge R, Pehlivan FZ, Isikli S (2020). The effect of positive childhood experiences on adult personality psychopathology. Personal Individ Differ.

[26] Schofield TJ, Conger RD, Neppl TK (2014). Positive parenting, beliefs about parental efficacy, and active coping: three sources of intergenerational resilience. J Fam Psychol.

[27] Dixon L, Browne K, Hamilton-Giachritsis C (2005). Risk factors of parents abused as children: a mediational analysis of the intergenerational continuity of child maltreatment (part I). J Child Psychol Psychiatry.

[28] Lev–Wiesel R. Intergenerational transmission of trauma across three generations: a preliminary study. Qual Soc Work 2007;6(1):75–94, DOI: 10.1177/1473325007074167.

[29] Murphy A, Steele M, Dube SR, Bate J, Bonuck K, Meissner P, et al. Adverse childhood experiences (ACEs) questionnaire and adult attachment interview (AAI): implications for parent child relationships. Child Abuse Negl. 2014;38(2):224–33. 10.1016/j.chiabu.2013.09.004. 10.1016/j.chiabu.2013.09.004 24670331

[30] Paclikova K, Veselska ZD, Bobakova DF, Palfiova M, Geckova AM (2019). What role do family composition and functioning play in emotional and behavioural problems among adolescent boys and girls?. Int J Public Health.

[31] Taylor SM, Ward P, Zabriskie R, Hill B, Hanson C (2012). Influences on active family leisure and a healthy lifestyle among adolescents. Leis Sci.

[32] CDC CfDCaP. Adverse Childhood Experiences (ACEs) 2016 [Updated 2020, April 3; cited July 31, 2020] Available from: https://www.cdc.gov/violenceprevention/acestudy/.

[33] Scott J (2006). Family and gender roles: how attitudes are changing. Arxius de Ciències Socials.

[34] Sacks V, Murphey D. The Prevalence of Adverse Childhood Experiences, Nationally, by State, and by Race or Ethnicity. Bethesda: Child Trends. 2018. www.childtrends.org/publications/prevalence-adverse-childhood-experiences-nationally-state-race-ethnicity. Accessed 1 Apr 2021.

[35] Bruner C (2017). ACE, place, race, and poverty: building hope for children. Acad Pediatr.

[36] Merrick JS, Narayan AJ, Atzl VM, Harris WW, Lieberman AF (2020). Type versus timing of adverse and benevolent childhood experiences for pregnant women’s psychological and reproductive health. Children Youth Services Review..

[37] Heim C, Plotsky PM, Nemeroff CB (2004). Importance of studying the contributions of early adverse experience to neurobiological findings in depression. Neuropsychopharmacology..

[38] Harnois G, Gabriel P, Organization WH (2000). Mental health and work: impact, issues and good practices.

[39] Shaw BA, Krause N, Chatters LM, Connell CM, Ingersoll-Dayton B (2003). Social structural influences on emotional support from parents early in life and adult health status. Behav Med.

[40] Price-Robertson R, Smart D, Bromfield L (2010). Family is for life connections between childhood family experiences and wellbeing in early adulthood. Fam Matters.

[41] Crouch E, Radcliff E, Strompolis M, Srivastav A (2019). Safe, stable, and nurtured: protective factors against poor physical and mental health outcomes following exposure to adverse childhood experiences (ACEs). J Child Adolesc Trauma.

[42] Leon K, Jacobvitz DB (2003). Relationships between adult attachment representations and family ritual quality: a prospective, longitudinal study. Fam Process.

[43] Crandall A, Broadbent E, Stanfill M, Magnusson BM, Novilla ML, Hanson CL, et al. The influence of adverse and advantageous childhood experiences during adolescence on young adult health. Child Abuse Negl. 2020;108:104644. 10.1016/j.chiabu.2020.104644. 10.1016/j.chiabu.2020.104644 32795716

[44] Dishion TJ, Shaw D, Connell A, Gardner F, Weaver C, Wilson M (2008). The family check-up with high-risk indigent families: preventing problem behavior by increasing parents’ positive behavior support in early childhood. Child Dev.

[45] Sege RD, Browne CH (2017). Responding to ACEs with HOPE: health outcomes from positive experiences. Acad Pediatr.

[46] Anda RF, Porter LE, Brown DW (2020). Inside the adverse childhood experience score: strengths, limitations, and misapplications. Am J Prev Med.

[47] Roulin N. Don't throw the baby out with the bathwater: comparing data quality of crowdsourcing, online panels, and student samples. Ind Organ Psychol. 2015;8(2):190–6. 10.1017/iop.2015.24.

[48] Walter SL, Seibert SE, Goering D, O’Boyle EH (2019). A tale of two sample sources: do results from online panel data and conventional data converge?. J Bus Psychol.

